# The Benefits of High-Intensity Interval Training on Cognition and Blood Pressure in Older Adults With Hypertension and Subjective Cognitive Decline: Results From the Heart & Mind Study

**DOI:** 10.3389/fnagi.2021.643809

**Published:** 2021-04-15

**Authors:** Narlon C. Boa Sorte Silva, Andrea F. M. Petrella, Nathan Christopher, Catherine F. S. Marriott, Dawn P. Gill, Adrian M. Owen, Robert J. Petrella

**Affiliations:** ^1^Department of Physical Therapy, Faculty of Medicine, University of British Columbia, Vancouver, BC, Canada; ^2^Centre for Studies in Family Medicine, Department of Family Medicine, Schulich School of Medicine and Dentistry, Western University, London, ON, Canada; ^3^The Brain and Mind Institute, Department of Physiology and Pharmacology and Department of Psychology, Western University, London, ON, Canada; ^4^School of Kinesiology, Faculty of Education, University of British Columbia, Vancouver, BC, Canada; ^5^Department of Family Practice, Faculty of Medicine, University of British Columbia, Vancouver, BC, Canada

**Keywords:** exercise, aging, elderly, cardiovascular risk, memory impairment

## Abstract

**Background:** The impact of exercise on cognition in older adults with hypertension and subjective cognitive decline (SCD) is unclear.

**Objectives:** We determined the influence of high-intensity interval training (HIIT) combined with mind-motor training on cognition and systolic blood pressure (BP) in older adults with hypertension and SCD.

**Methods:** We randomized 128 community-dwelling older adults [age mean (SD): 71.1 (6.7), 47.7% females] with history of hypertension and SCD to either HIIT or a moderate-intensity continuous training (MCT) group. Both groups received 15 min of mind-motor training followed by 45 min of either HIIT or MCT. Participants exercised in total 60 min/day, 3 days/week for 6 months. We assessed changes in global cognitive functioning (GCF), Trail-Making Test (TMT), systolic and diastolic BP, and cardiorespiratory fitness.

**Results:** Participants in both groups improved diastolic BP [*F*_(1, 87.32)_ = 4.392, *p* = 0.039], with greatest effect within the HIIT group [estimated mean change (95% CI): −2.64 mmHg, (−4.79 to −0.48), *p* = 0.017], but no between-group differences were noted (*p* = 0.17). Both groups also improved cardiorespiratory fitness [*F*_(1, 69)_ = 34.795, *p* < 0.001], and TMT A [*F*_(1, 81.51)_ = 26.871, *p* < 0.001] and B [*F*_(1, 79.49)_ = 23.107, *p* < 0.001]. There were, however, no within- or between-group differences in GCF and systolic BP at follow-up.

**Conclusion:** Despite improvements in cardiorespiratory fitness, exercise of high- or moderate-intensity, combined with mind-motor training, did not improve GCF or systolic BP in individuals with hypertension and SCD.

**Clinical Trial Registration:** ClinicalTrials.gov (NCT03545958).

## Introduction

Hypertension is associated with cognitive impairment in older adults (Iadecola et al., 2016), contributing heavily to cerebrovascular (Dichgans and Leys, 2017) and Alzheimer's disease pathophysiology (Rodrigue et al., 2013). Healthy older adults with subjective cognitive decline (SCD) may experience subtle cognitive deterioration due to brain pathology accumulating years before clinical diagnoses (Buckley et al., 2015). Individuals with history of both hypertension and SCD may be at higher risk of dementia because of increased cerebrovascular disease and neurodegenerative burden (Uiterwijk et al., 2014). Despite greater risk, the effects of non-pharmacological interventions to ameliorate cognition in these individuals remain unknown.

Exercise has been associated with improved cognition (Lautenschlager et al., 2008), and has been shown to positively impact both brain function (Voss et al., 2010) and structure (Erickson et al., 2011) in older adults, but evidence is limited in those with hypertension (Smith et al., 2010). High-intensity interval training (HIIT) is a modality of exercise training that yields similar or greater cardiorespiratory fitness improvements compared to conventional, moderate-intensity continuous training (MCT) (Wisløff et al., 2007). In clinical populations, HIIT has been shown to lower blood pressure (BP) to a greater extent compared to MCT, including within hypertensive patients (Pescatello et al., 2015). The effects of HIIT on cognition in older adults is understudied, as most trials have employed only MCT protocols (Northey et al., 2017). Further, with growing interest on multidomain interventions to improve cognition (Kivipelto et al., 2018), combining HIIT with mind-motor training approaches seems appealing. Square-stepping exercise (SSE) (Shigematsu et al., 2008) is a novel type of mind-motor training associated with positive effects on cognition (Gill et al., 2016); however, SSE has yet to be studied in individuals with hypertension and SCD.

In this study, we investigated the effects of combining HIIT with SSE on cognition and BP in older adults with a history of hypertension and SCD. We hypothesized that HIIT plus SSE would yield superior improvements in both BP and cognition outcomes compared to an active control group.

## Methods

### Study Design and Participants

We conducted a 6-month, single-blind, two-arm randomized controlled trial based in the community following a pragmatic approach (Ford and Norrie, 2016). Participants were randomized to either intervention (HIIT) or comparator (MCT) groups. Randomization (1:1) was conducted via www.randomization.com with randomly selected block sizes (e.g., 4, 6, 8) (Friedman et al., 2010). Block randomization was used to avoid statistical challenges posed by clustering with simple randomization or sample size imbalance and loss of power (Friedman et al., 2010), while ensuring similar sample sizes in both groups at every 4, 6, or 8 blocks. Each participant had a 50% chance of being randomized to either group. No demographic characteristics or other factors were considered in the randomization procedure.

We included individuals who met the following criteria: (1) 55 years of age or older; (2) presented with a history of controlled or uncontrolled stage 1 hypertension, or taking antihypertensive BP medication (Leung et al., 2017); (3) had preserved instrumental activities of daily living (scoring >6/8 on the Lawton-Brody Instrumental Activities of Daily Living scale (Lawton and Brody, 1969)]; (4) presented with signs of SCD (defined as answering yes to the question: “Do you feel like your memory or thinking skills have gotten worse recently?”), as employed in previous exercise studies (Barnes et al., 2013); (5) preserved objective cognitive performance defined by scoring ≥26 on the Montreal Cognitive Assessment (MoCA) (McLennan et al., 2011) combined with study physician consult; and (6) able to comprehend the study letter of information and provide written informed consent.

Participants were excluded if they presented with: (1) significant neurological conditions or psychiatric disorders (e.g., diagnosis of Alzheimer's disease or vascular dementia, Parkinson's disease, stroke <1 year prior); (2) history of severe cardiovascular conditions [e.g., recent (<1 year) myocardial infarction] or symptomatic cerebrovascular disease; (3) significant orthopedic conditions; or (4) untreated clinical depression (i.e., score >15 on the Centers for Epidemiologic Studies Depression scale (Lewinsohn et al., 1997) combined with study physician consult). Participants were also excluded for any other factors that could potentially limit their ability to fully participate in the study.

All research participants provided written informed consent prior to partaking in any of the research activities. The Western Health Sciences Research Ethics Board approved the study and the trial was registered within ClinicalTrials.gov (NCT03545958) on May 22, 2018.

### Interventions

Participants in both groups engaged in a 60-min, group-based, combined exercise program. Each session began with 15 min of mind motor training (i.e., SSE) followed by 45 min of either HIIT or MCT. The interventions were conducted with ≤30 participants/session, 3 days/week on non-consecutive days, for 6 months at the local YMCA. Participants used stationary bikes during HIIT or MCT and were coached by qualified fitness instructors with student volunteer assistance. Exercise intensity during HIIT and MCT was prescribed individually using training heart rate (HR), determined via exercise testing (see “*Cardiorespiratory fitness”* subsection) (American College of Sports Medicine, 2014). Intensity was monitored using chest-based HR monitors with a group tracking system (Myzone™) and via the modified 10-point Borg Rating of Perceived Exertion (RPE) scale (Borg, 1982). To increase motivation and ensure compliance with intensity protocol, each participant individual HR and % of HRmax achieved were continuously displayed to participants on a large screen during the exercise sessions. Intensity was stimulated via continuous increments in speed and/or resistance on the stationary bikes.

#### Mind-Motor Training

**Figure 1:**
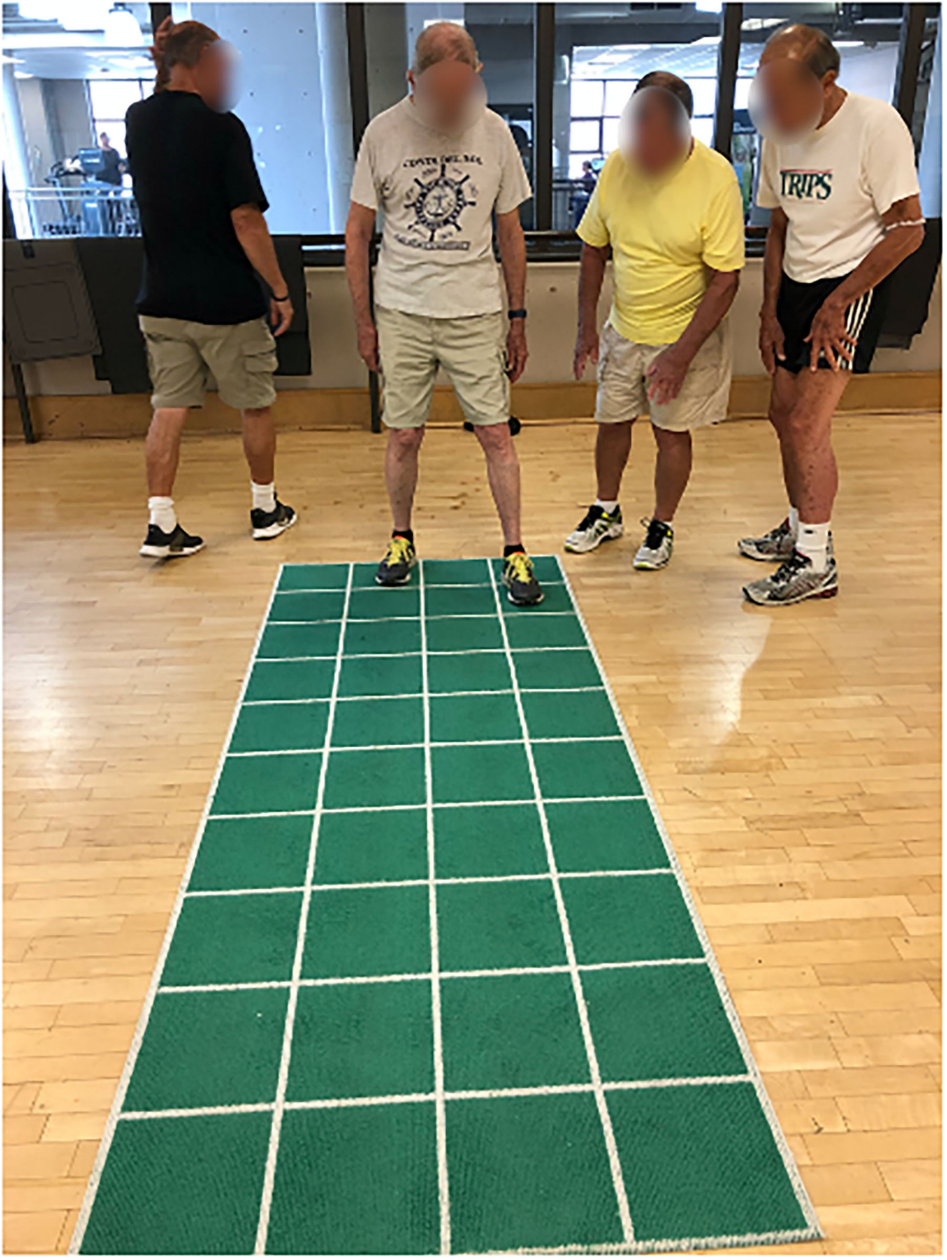
Participants performing the square-stepping exercise.

The SSE program is a group-based, visuospatial working memory task with a stepping response (see Figure 1) (Shigematsu et al., 2008). The training protocol entails the reproduction of complex stepping patterns on a gridded floor mat (2.5 m × 1 m). The stepping patterns are demonstrated by an instructor and participants are expected to memorize and then attempt to reproduce each pattern. The complexity of these patterns is based on the number of steps, order and direction of foot placement on the mat. Complexity was increased gradually in each session with the introduction of new patterns once 80% of participants had learned and repeated each pattern twice. The SSE sessions were conducted in groups ≤6 participants/mat, and participants were encouraged to assist each other by providing visual and verbal cues.

#### High-Intensity Interval Training

Each HIIT session was composed of a 5 to 10-min warm-up, a 25-min main activity, and a 5 to 10-min cool down. The 25-min main activity included 4 bouts of different exercise intensities (Molmen-Hansen et al., 2012). In each bout, participants received 4 min of high-intensity cycling (starting at 80–90% HRmax, and progressing toward 85–95% HRmax) followed by 3 min of active rest (aiming for 40–60% HRmax) (Molmen-Hansen et al., 2012). This HIIT protocol was deemed safe to be implemented in our study as it was originally designed to reduce systolic (SBP) in patients with essential hypertension at baseline (Molmen-Hansen et al., 2012), and has been safely applied to several other clinical populations (Mezzani et al., 2012). Exercise intensity was monitored throughout the HIIT sessions with volunteers collecting HR and RPE once during warm-up and cool-down, as well as during each active and rest period. Participants were verbally encouraged by instructors to achieve the prescribed exercise intensity in each high-intensity, 4-min active period. Progression was made gradually over the course of the study during each session until participants were able to comfortably reach and/or maintain the high-intensity training zone (i.e., 80–95% HRmax) during the active high-intensity periods.

#### Moderate-Intensity Continuous Training

Each MCT session consisted of a 5 to 10-min warm-up, a 25-min continuous cycling at moderate-intensity (starting at 60–80% HRmax) component, and a 5 to 10-min cool down. Exercise intensity was monitored throughout the MCT sessions with volunteers collecting HR and RPE once during warm-up and cool-down, and every 5 min within the 25-min main component. Participants were also encouraged verbally by instructors to maintain their HR within the moderate-intensity training zone during each exercise session (i.e., 60–80% HRmax).

### Measurements

Participants attended baseline measurements prior to study randomization (i.e., blind baseline assessments). We collected clinical and demographic information, and performed cardiovascular, cardiorespiratory and cognitive assessments over 2 days. Baseline and 6-month follow-up measurements were conducted by trained assessors; at follow-up, assessors were blinded to group allocation for assessment of BP and cognition. All follow-up measurements were performed within 1–2 weeks after the end of the study intervention.

#### Cognition Assessment

Global cognitive functioning (GCF) was the primary outcome of this study, derived from the Cambridge Brain Sciences (CBS) battery (Hampshire et al., 2012). The CBS battery contains 12 non-verbal, culturally independent cognitive tasks covering four broad cognitive domains (i.e., memory, reasoning, concentration, and planning, see details in Supplementary Methods) (Hampshire et al., 2012). It is a fully automated, computerized adaptive testing platform and has been used to effectively evaluate cognition in several large-scale studies (Wild et al., 2018). The tasks were conducted using laptops with access to internet provided by study personnel. Before each task, participants practiced with a tutorial under the guidance of a trained assessor. Assessors provided assistance as necessary but were instructed not to intervene once participants completed the tutorial and data collection began. The outcome measure was derived based on participant scores on each of the 12 CBS tasks. Following previous methods (Gill et al., 2016), task scores were z-transformed and averaged to create domain-specific composite scores. These composite scores were then averaged to create a GCF score, used as the primary outcome. Domain-specific scores were retained for secondary analysis. Additionally, we administered the paper-based Trail-Making Test (TMT) to assess changes in lower-level cognitive processes (e.g., processing speed, set-shifting) (Reitan, 1992). This measure was included since it is a commonly used test in exercise studies due to its sensitivity to capture the effects of exercise on cognition in older adults (Boa Sorte Silva et al., 2020a).

#### Blood Pressure Assessment

Automated office SBP was the co-primary outcome in this study. Measurements were obtained by trained technicians with an automated monitor (Watch BP Office, Microlife AG, Switzerland), with standardized cuff size and position. The left arm was preferred for BP assessments across all participants whenever possible (i.e., exceptions were severe discomfort caused by muscle injuries or scar tissue from previous surgeries). Nonetheless, the same arm was used for both baseline and follow-up assessments to ensure consistency within participants. After a 5-min seated resting period, BP was collected four times with 1-min intervals (Leung et al., 2017). The first BP measurement was discarded and the average of the last three SBP readings were used for analysis. We also retained the last three measures of diastolic BP (DBP) and resting HR as secondary outcomes. We performed BP measurements within 1–2 weeks after the end of the study intervention but no earlier than 24 h after the last exercise session attended by the study participants. Furthermore, to minimize the influence of other external factors, participants were asked to take their medication as usual, refrain from alcohol consumption and vigorous exercise 24 h prior to assessment, refrain from caffeine intake on the day of assessment, and were asked void their bladder before BP measurements.

#### Cardiorespiratory Fitness

We conducted maximal exercise testing (Gibbons et al., 1997) using a treadmill (Quinton® TM55) connected to a desktop computer equipped with the Q-Stress™ Cardiac Science™ software, Version 4.5 (Cardiac Science Corp., USA). The maximal test ended once participants had subjectively reached their maximum capacity and asked for the test to be stopped. The test was also ended under the study physician's recommendations. We monitored HR via exercise echocardiogram (ECG) with electrodes (10-lead) connected to participant's chest. The Bruce (Bruce et al., 1973) treadmill test was applied in this study and we retained time to exhaustion, estimated metabolic equivalent (MET), and HRmax for secondary outcome analysis.

### Samples Size

A meta-analysis (Colcombe and Kramer, 2003) suggested that exercise improves cognition with an overall effect size of *d* = 0.48. Further, Cornelissen and Fagard (2005) reported that exercise training is associated with an overall 6.9 mmHg reduction in SBP hypertensive patients, with an effect size of *d* = 0.85 (Morris, 2008). Considering a greater effect of exercise on SBP, we estimated our sample size using an approximate effect size for cognition. Based on this, with 63 participants per group, our study would have 80% power at the 5% significance level to detect a moderate effect size of *d* = 0.55 (Lachin, 1981). We estimated a dropout rate of 20% during the 6-month period, which increased our calculation to 70 participants per group. This proposed sample size is in line with previous investigations (Lautenschlager et al., 2008).

### Statistical Analyses

We analyzed the outcome data based on an intent-to-treat approach, using linear mixed effects regression models for repeated measurements (LMM) (Fitzmaurice et al., 2004). We included all randomized participants, regardless of missing data at follow-up (Fitzmaurice et al., 2004). Time was considered as a repeated, categorical variable included as a fixed effect in addition to group and group-by-time interaction (Fitzmaurice et al., 2004). For primary outcomes, we examined the difference between groups in estimated mean change in GCF and SBP from baseline to 6 months.

For our secondary outcomes, we examined changes in domain-specific cognition (i.e., memory, reasoning, concentration, and planning), TMT parts A and B, cardiovascular outcomes (i.e., office DBP and resting HR), and cardiorespiratory fitness (i.e., time to exhaustion, METs achieved and HRmax). We also conducted sensitivity analyses adjusting for age, sex, MoCA scores, baseline cardiorespiratory fitness (i.e., time to exhaustion), as well as history and/or medication use for diabetes, cardiovascular disease (including hypertension), and depression.

Furthermore, to handle missing data and perform confirmatory analysis in both primary outcomes, we first conducted complete-case analysis including only participants who completed baseline and 6-month assessment. Second, we used multiple imputation under the assumption that data were missing at random [Little's MCAR test: χ^2^(137) = 159.99, *p* = 0.087]. Following previous methods (Ten Brinke et al., 2020), we created 40 imputed datasets using random number generation and repeated the LMM for both GCF and SBP. The results of each LMM were pooled across all imputed datasets.

Additionally, we used *post-hoc* analyses to investigate subgroup effects on primary outcomes based on sex, and baseline median values for SBP and cardiorespiratory fitness (i.e., time to exhaustion). We based our interpretation of study results on estimation and associated 95% confidence intervals (CI). For all subgroup analyses, we adjusted *p* using the Benjamini-Hochberg false discovery rate approach (Benjamini and Hochberg, 1995), with an adjusted significance threshold *p* ≤ 0.005.

Analyses were performed on IBM® SPSS® Statistics, Version 24 (IBM Corp, USA), and R, Version 3.6.1 (http://www.R-project.org).

## Results

### Enrollment and Adherence

**Figure 2:**
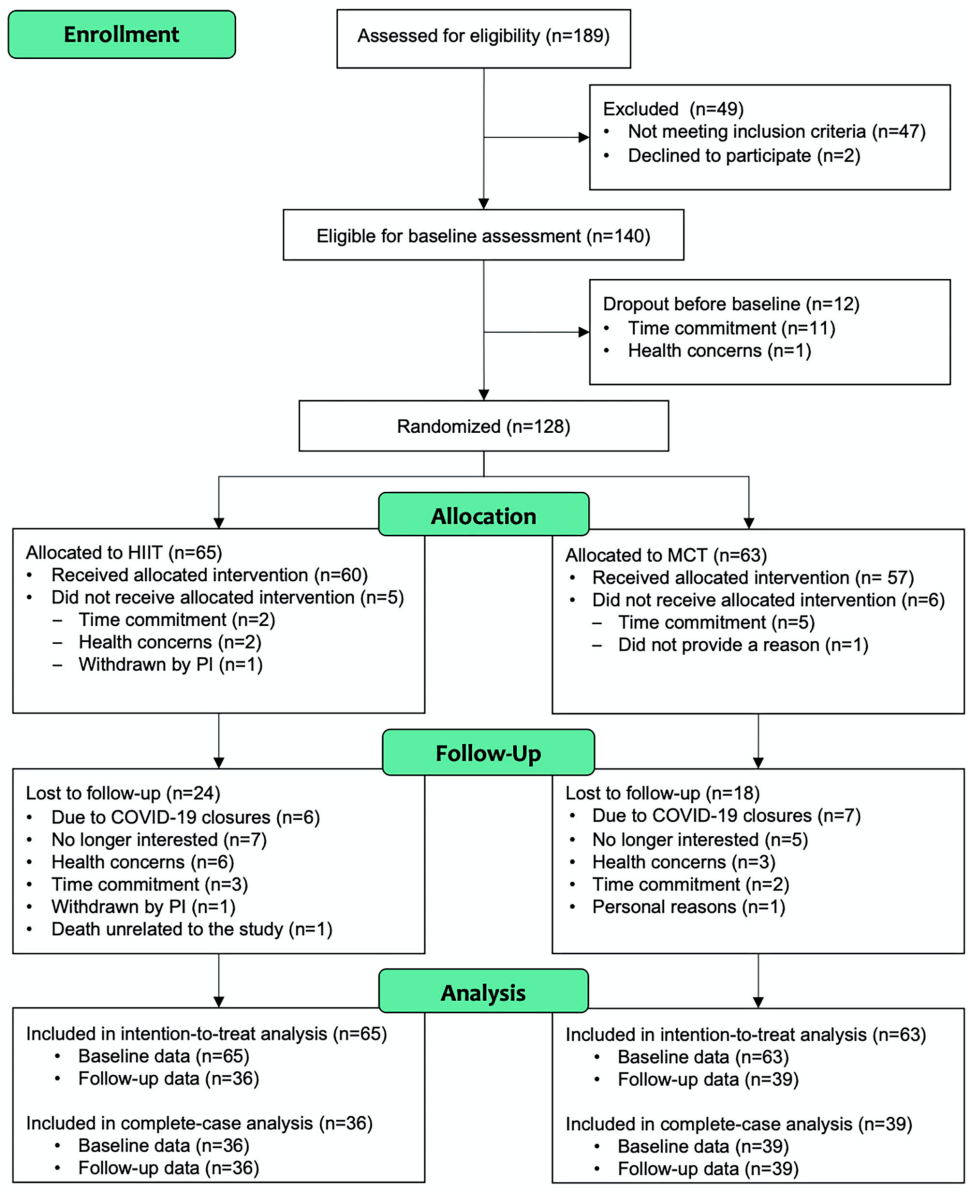
Study CONSORT diagram to illustrate participant flow throughout the study.

Participant flow during the study is shown Figure 2. The study period ran between July 2018 and March 2020. Participant demographic information and clinical characteristics are presented in Table 1, while baseline outcome measures are reported in Table 2. Overall, at baseline, participants included (*n* = 128) had preserved cognitive function (McLennan et al., 2011), were mostly Caucasian, highly educated (Table 1), and had “greater than average” cardiorespiratory fitness (Mandsager et al., 2018) compared to normative data (see Table 2). Average attendance to the exercise sessions was 54% for the MCT group and 51% for the HIIT group, with no differences between groups [*t*_(126)_ = 0.45, *p* = 0.65].

**Table 1:** Participant demographics and clinical characteristics.

**Baseline descriptors^a^**	**HIIT (*n* = 65)**	**MCT (*n* = 63)**	**Total (*n* = 128)**	***p*-value^b^**
Demographics				
Age, yr	71.7 (6.3)	70.4 (7.1)	71.1 (6.7)	0.27
Females, *n* (%)	32 (49.2)	29 (46.0)	61 (47.7)	0.85
Caucasian, *n* (%)	56 (86.2)	57 (90.5)	113 (88.3)	0.76
Education, yr	16 (3.2)	16.6 (3.6)	16.3 (3.4)	0.38
MoCA, score	26.9 (1.6)	26.8 (1.5)	26.8 (1.6)	0.81
MMSE, score	29.2 (1.1)	29.2 (1.0)	29.2 (1.0)	0.96
CES-D, score	9.0 (7.3)	8.8 (7.1)	8.9 (7.2)	0.83
IADL	7.9 (0.3)	7.9 (0.3)	7.9 (0.3)	
Height, cm	166.7 (9.2)	167.7 (11.3)	167.2 (10.3)	0.57
Weight, kg	81.5 (17.1)	85.4 (21.5)	83.4 (19.4)	0.25
BMI, kg/m^2^	29.3 (5.8)	30.3 (6.5)	29.8 (6.2)	0.38
Diagnosed comorbidities, *n* (%)				
Hypertension	51 (78.5)	49 (77.8)	100 (78.1)	1.00
Arthritis	23 (35.4)	25 (39.7)	48 (37.5)	0.75
Diabetes	11 (16.9)	13 (20.6)	24 (18.8)	0.76
Depression	4 (6.2)	12 (19.0)	16 (12.5)	0.053
Medication usage, *n* (%)				
Blood pressure	53 (81.5)	53 (84.1)	106 (82.8)	0.88
Cholesterol	31 (47.7)	33 (52.4)	64 (50.0)	0.72
Diabetes	9 (13.8)	13 (20.6)	22 (17.2)	0.43
Depression	6 (9.2)	11 (17.5)	17 (13.3)	0.27
Arthritis	6 (9.2)	5 (7.9)	11 (8.6)	1.00
Blood thinners	3 (4.6)	4 (4.8)	7 (5.5)	0.97

a *Data presented as mean (standard deviation) unless otherwise indicated*.

b *Significance for independent samples t-test for continuous variables, or Chi-square test for independence for categorical variables. HIIT, high-intensity interval training; MCT, moderate-intensity continuous training; MoCA, Montreal Cognitive Assessment; MMSE, Mini-Mental State Examination; CES-D, Center for Epidemiologic Studies Depression Scale; IADL, Instrumental Activities of Daily Living Scale*.

**Table 2:** Study baseline measures.

**Baseline outcomes^a^**	**HIIT (*n* = 65)**	**MCT (*n* = 63)**	**Total (*n* = 128)**	***p-*value^b^**
Cognition, z score				
GCF	0.004 (0.553)	−0.004 (0.594)	0 (0.571)	0.94
Memory	−0.03 (0.609)	0.031 (0.695)	0 (0.651)	0.60
Concentration	−0.01 (0.745)	0.01 (0.752)	0 (0.746)	0.88
Planning	0.031 (0.791)	−0.032 (0.839)	0 (0.812)	0.66
Reasoning	0.023 (0.668)	−0.024 (0.739)	0 (0.702)	0.71
Blood pressure, mm Hg				
Systolic	129.8 (16.0)	128.8 (16.0)	129.3 (15.9)	0.73
Diastolic	73.3 (8.3)	72.8 (8.7)	73.1 (8.5)	0.75
Trail-Making Test, s, median (IQR)				
Part A	36.0 (28.0, 44.0)	32.0 (28.0, 41.7)	34.5 (28.0, 43.0)	0.39
Part B	65.0 (49.0, 94.5)	68.0 (49.0, 92.0)	65.2 (49.0, 92.75)	0.81
Cardiorespiratory fitness^c^				
Time to exhaustion, min	7.0 (2.3)	6.9 (2.2)	6.9 (2.2)	0.93
Maximum intensity, METs	8.6 (2.4)	8.6 (2.2)	8.6 (2.3)	0.97
Maximum heart rate, bpm	144.2 (18.4)	144.9 (18.8)	144.5 (18.5)	0.85

a *Data presented as mean (standard deviation) unless otherwise indicated*.

b *Significance for independent samples t-test for continuous variables*.

c *Exercise stress test data available from 118 (HIIT = 62, MCT = 56). HIIT, high-intensity interval training; MCT, moderate-intensity continuous training; GCF, global cognitive functioning; IQR, interquartile range; METs, metabolic equivalent*.

**Figure 3:**
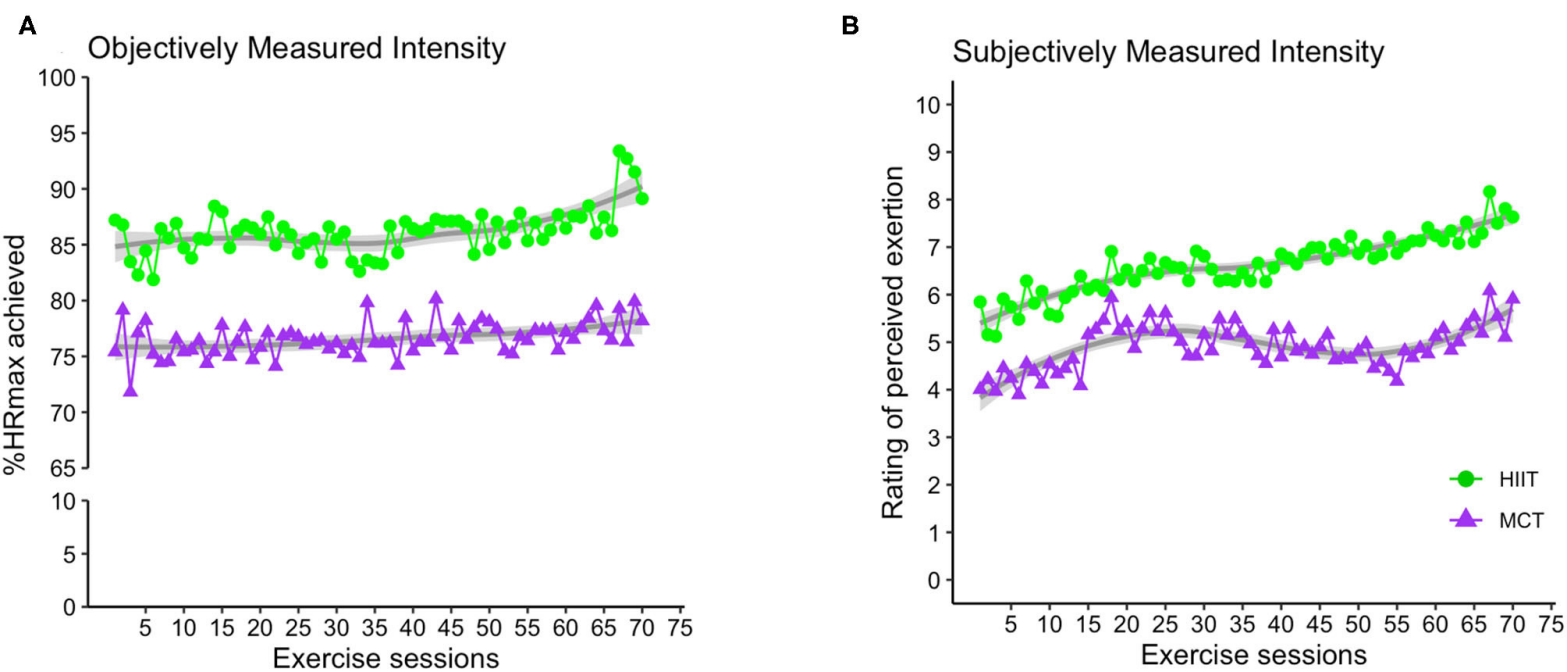
Group average for exercise intensity achieved over the course of the exercise sessions as indexed by %HRmax **(A)** and RPE **(B)**. For the HIIT group, data shown represent average exercise intensity at the 2-min mark within each 4-min bout of HIIT exercise. For MCT group, the data indicate average exercise intensity collected at 4 different timepoints within the each MCT session (i.e., at 5, 10, 20, and 25 min during the main MCT component, i.e., after 5 to 10 min of warm-up). %HRmax, percentage of maximum heart rate; RPE, rating of perceived exertion; HIIT, high-intensity interval training; MCT, moderate-intensity continuous training.

Adherence to exercise intensity was monitored using %HRmax and RPE data collected during the exercise sessions. Figure 3 demonstrates average exercise intensity for both HIIT and MCT groups in each exercise session. The data indicate that both groups exercised at different intensities as prescribed in the study protocol, with participants in the HIIT group exercising at higher intensities [mean (standard deviation), %HRmax = 85.01 (6.93), RPE = 6.29 (1.24)] compared to those in the MCT group [%HRmax = 75.91 (6.28), RPE = 4.68 (1.20)]. An independent samples *t*-test comparing average exercise intensity achieved by each participant across all exercise sessions revealed that indeed the HIIT group performed at higher intensity as indexed by %HRmax [*t*_(110)_ = 7.28, *p* < 0.001] and RPE [*t*_(110)_, = 7.00, *p* < 0.001] data collected.

### Outcomes

Main results (intention-to-treat approach) are shown in Table 3. Complete-case and multiple-imputation data analysis revealed similar results (see Supplementary Table 1).

**Table 3:** Within- and between-group differences from baseline to 6 months by randomization group.

		**Within-group estimated mean change (95% CI)**				**Between-group differences (95% CI)**	
**Outcomes^a^**		**HIIT (*n* = 65)**	***p* Value**	**MCT (*n* = 63)**	***p* Value**	**6 months (*n* = 128)**	***p* Value**
Cognition, z score							
GCF							
	Unadjusted	−0.02 (−0.12 to 0.08)	0.67	0.06 (−0.04 to 0.16)	0.23	−0.08 (−0.22 to 0.06)	0.25
	Adjusted	−0.03 (−0.14 to 0.08)	0.64	0.04 (−0.07 to 0.14)	0.48	−0.06 (−0.21 to 0.08)	0.38
Memory							
	Unadjusted	−0.02 (−0.18 to 0.14)	0.83	0.12 (−0.03 to 0.28)	0.11	−0.14 (−0.36 to 0.08)	0.20
	Adjusted	−0.004 (−0.17 to 0.16)	0.96	0.11 (−0.05 to 0.27)	0.19	−0.11 (−0.34 to 0.11)	0.31
Concentration						
	Unadjusted	−0.10 (−0.29 to 0.08)	0.28	0.08 (−0.10 to 0.25)	0.40	−0.18 (−0.43 to 0.08)	0.18
	Adjusted	−0.14 (−0.33 to 0.06)	0.18	0.05 (−0.15 to 0.24)	0.63	−0.18 (−0.45 to 0.08)	0.17
Planning							
	Unadjusted	0.02 (−0.21 to 0.25)	0.89	−0.04 (−0.26 to 0.18)	0.69	0.06 (−0.26 to 0.38)	0.71
	Adjusted	−0.04 (−0.28 to 0.21)	0.77	−0.04 (−0.28 to 0.20)	0.75	0.002 (−0.33 to 0.33)	0.99
Reasoning							
	Unadjusted	0.06 (−0.16 to 0.27)	0.60	0.14 (−0.06 to 0.35)	0.18	−0.08 (−0.38 to 0.21)	0.57
	Adjusted	0.05 (−0.18 to 0.27)	0.68	0.10 (−0.12 to 0.32)	0.39	−0.05 (−0.35 to 0.26)	0.76
Blood pressure							
Systolic, mmHg							
	Unadjusted	−2.58 (−6.62 to 1.46)	0.21	1.16 (−2.76 to 5.07)	0.56	−3.74 (−9.36 to 1.89)	0.19
	Adjusted	−0.24 (−4.52 to 4.04)	0.91	2.86 (−1.32 to 7.06)	0.18	−3.11 (−8.84 to 2.62)	0.28
Diastolic, mmHg							
	Unadjusted	**−2.64 (−4.79 to** –**0.48)**	**0.017**	−0.53 (−2.63 to 1.56)	0.62	−2.11 (−5.11 to 0.90)	0.17
	Adjusted	**−2.41 (−4.69 to** –**0.12)**	**0.039**	−0.47 (−2.71 to 1.78)	0.68	−1.94 (−5.01 to 1.13)	0.21
Resting heart rate, bpm							
	Unadjusted	−1.76 (−4.81 to 1.29)	0.26	0.54 (−2.40 to 3.48)	0.72	−2.3 (−6.53 to 1.94)	0.28
	Adjusted	−1.35 (−4.48 to 1.77)	0.39	1.19 (-1.86 to 4.24)	0.44	−2.54 (−6.72 to 1.64)	0.23
Trail-Making Test^b^							
Part A							
	Unadjusted	**−0.07 (−0.11 to** **−0.03)**	**<0.001**	**−0.07 (−0.10 to** **−0.03)**	**0.001**	−0.005 (−0.06 to 0.05)	0.86
	Adjusted	**−0.06 (−0.10 to** **−0.02)**	**<0.003**	**−0.07 (−0.11 to** **−0.03)**	**<0.001**	0.01 (−0.04 to 0.07)	0.69
Part B							
	Unadjusted	**−0.07 (−0.11 to** **−0.03)**	**0.001**	**−0.06 (−0.09 to** **−0.02)**	**0.002**	−0.01 (−0.06 to 0.04)	0.68
	Adjusted	**−0.06 (−0.10 to** **−0.01)**	**<0.009**	**−0.05 (−0.09 to** **−0.005)**	**0.029**	−0.01 (−0.7 to 0.04)	0.69
B minus A							
	Unadjusted	−0.002 (−0.05 to 0.05)	0.93	0.001 (−0.05 to 0.05)	0.97	−0.003 (−0.07 to 0.07)	0.93
	Adjusted	0.01 (−0.04 to 0.07)	0.64	0.03 (−0.03 to 0.08)	0.31	−0.01 (−0.09 to 0.06)	0.69
Cardiorespiratory fitness^c^							
Time to exhaustion, min							
	Unadjusted	**1.12 (0.63 to 1.62)**	**<0.001**	**0.93 (0.45 to 1.42)**	**<0.001**	0.19 (−0.51 to 0.88)	0.59
	Adjusted	**1.14 (0.64 to 1.63)**	**<0.001**	**0.95 (0.46 to 1.44)**	**<0.001**	0.19 (−0.51 to 0.88)	0.59
Intensity, METs							
	Unadjusted	**1.18 (0.58 to 1.78)**	**<0.001**	**0.83 (0.24 to 1.43)**	**0.007**	0.35 (−0.50 to 1.20)	0.42
	Adjusted	**1.14 (0.54 to 1.74)**	**<0.001**	**0.83 (0.24 to 1.42)**	**0.006**	0.31 (−0.53 to 1.15)	0.47
Maximum heart rate, bpm							
	Unadjusted	−0.24 (−4.91 to 4.42)	0.92	0.65 (−3.95 to 5.25)	0.78	−0.89 (−7.44 to 5.66)	0.79
	Adjusted	−0.29 (−4.95 to 4.36)	0.90	0.64 (−3.96 to 5.24)	0.78	−0.93 (−7.48 o 5.61)	0.78

a *Calculated from linear mixed effects regression models that included group (HIIT or MCT), time (baseline and 6 months), and group × time interaction terms. Results are presented as intention-to-treat approach. Bold numbers indicate significant differences. Adjusted models account for the influence of age, sex, Montreal Cognitive Assessment score, baseline cardiorespiratory fitness, as well as history or medication use for diabetes, cardiovascular disease, and depression*.

b *Log10 transformation applied*.

c *Data available from 118 (HIIT = 62, MCT = 56). CI, confidence interval; HIIT, high-intensity interval training; MCT, moderate-intensity continuous training, GCF, global cognitive functioning; METs, metabolic equivalent*.

### Cognition

**Figure 4:**
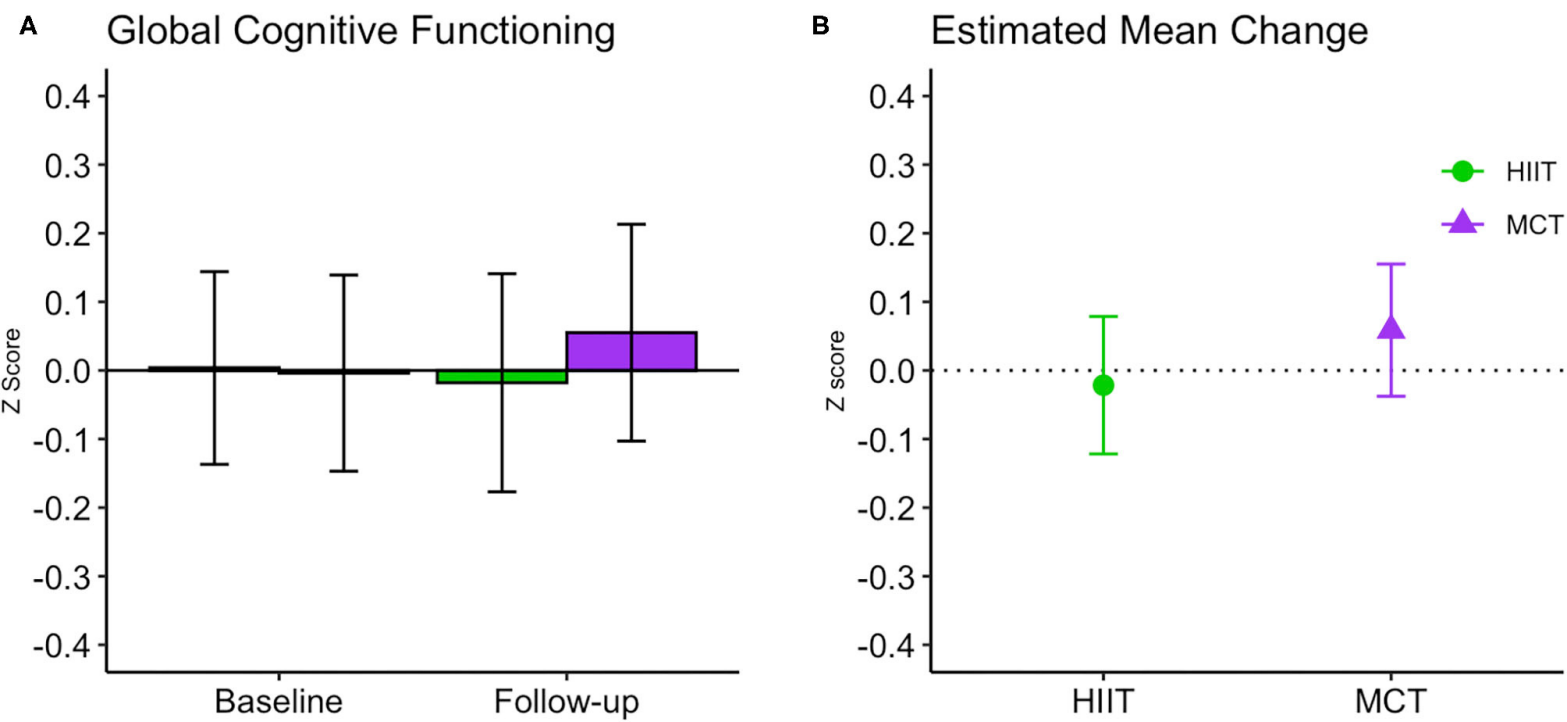
Baseline and follow-up means for global cognitive function (**A**, group mean and 95% CI) and estimated mean change from baseline to 6 months (**B**, estimated mean change and 95% CI). CI, confidence interval; HIIT, high-intensity interval training; MCT, moderate-intensity continuous training.

**Figure 5:**
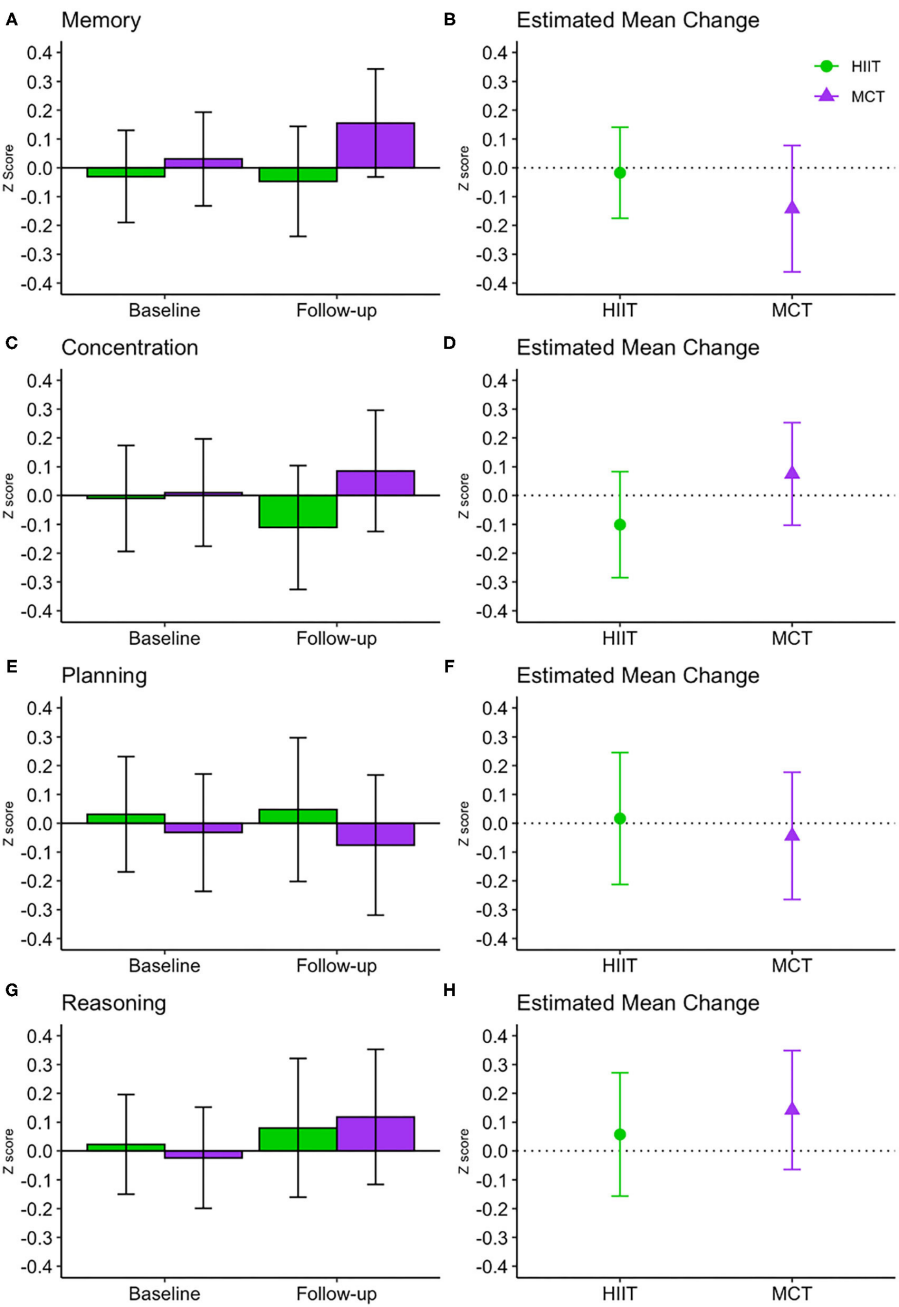
Baseline and follow-up means for domain-specific cognitive function (**A,C,E,G**, show group mean and 95% CI) and estimated mean change from baseline to 6 months (**B,D,F,H** show estimated mean change and 95% CI). CI, confidence interval; HIIT, high-intensity interval training; MCT, moderate-intensity continuous training.

There were no significant within- or between-group differences in GCF or any of the domain-specific composite scores at 6 months (see Table 3 and Figures 4, 5); sensitivity analyses indicated that results remained unchanged in fully adjusted models (Table 3). While there were no differences between groups, we did observe improvements in both groups in processing speed [*F*_(1, 81.51)_ = 26.871, *p* < 0.001] and mental flexibility/executive functioning [*F*_(1, 79.49)_ = 23.107, *p* < 0.001] measured using the paper-based TMT A and B, respectively. Nonetheless, the more complex TMT set-shifting score (Part A-B) did not change over time in either group (see Table 3). These findings for TMT A and B remained significant in fully adjusted models [Part A: *F*_(1, 89.65)_ = 21.572, *p* < 0.001, and Part B: *F*_(1, 86.84)_ = 10.947, *p* < 0.001].

### Blood Pressure

**Figure 6:**
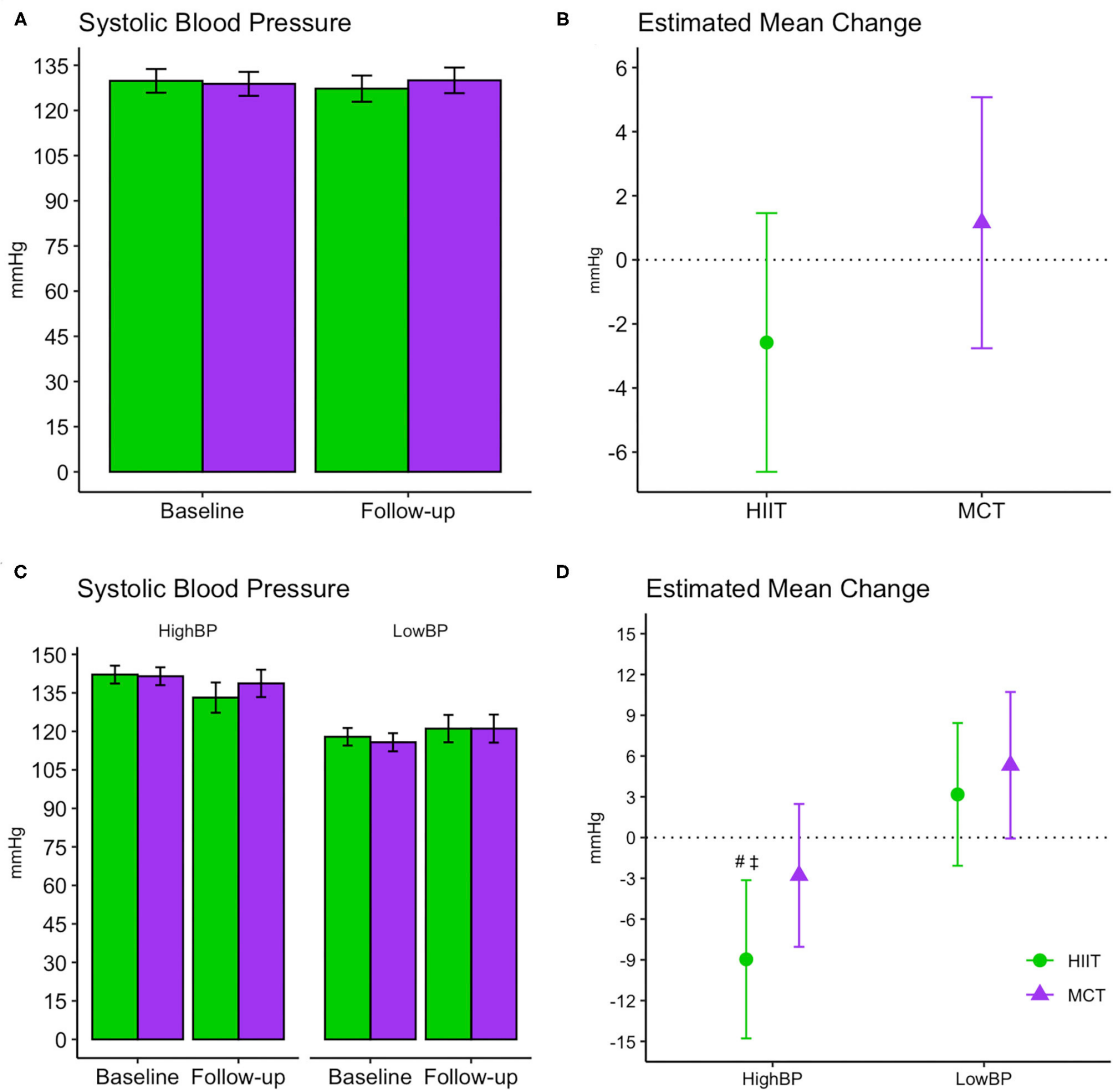
Baseline and follow-up means for systolic blood pressure readings (**A,C**, group mean and 95% CI) and estimated mean change from baseline to 6 months (**B,D**, estimated mean change and 95% CI). **(A,B)** Display changes for the entire sample, while **(C,D)** display changes within subgroups stratified by baseline systolic blood pressure readings. #, significant changes over time at adjusted significance threshold (*p* ≤ 0.005); See Supplementary Table 2 for further details. ‡, greater changes compared to HIIT Low BP [difference between groups (CI): −12.14 mmHg (−19.98 to −4.30), *p* = 0.003] and MCT Low BP [−14.28 mmHg (−22.22 to −6.35), *p* = 0.001] at adjusted significance threshold (*p* ≤ 0.005). CI, confidence interval; HIIT, high-intensity interval training; MCT, moderate-intensity continuous training; High BP, high systolic blood pressure at baseline; Low BP, low systolic blood pressure at baseline.

No significant changes were seen for SBP or resting HR and results remained unchanged in fully adjusted models. Both groups improved DBP at follow-up [*F*_(1, 87.32)_ = 4.392, *p* = 0.04], and while changes were driven by a greater reduction in the HIIT group, the between-group difference did not reach statistical significance. Change in DBP remained significant for the HIIT group [estimated mean change (95% CI): −2.41 mmHg, (−4.69 to −0.12), *p* = 0.039] in fully adjusted models (Table 3)]. We repeated our analysis excluding 9 participants who had changes in their BP medication and found that DBP changes remained significant within the HIIT group [estimated mean change (95% CI): −2.47 mmHg, (−4.83 to −0.10), *p* = 0.041, *n* = 119]. Further subgroup analyses revealed reduction in SBP for HIIT participants with high SBP at baseline (i.e., those with SBP ≥ 128 mmHg). This improvement was statistically superior compared to participants with low BP in both HIIT and MCT subgroups at the adjusted significance threshold (*p* ≤ 0.005), see Figure 6.

### Cardiorespiratory Fitness

**Figure 7:**
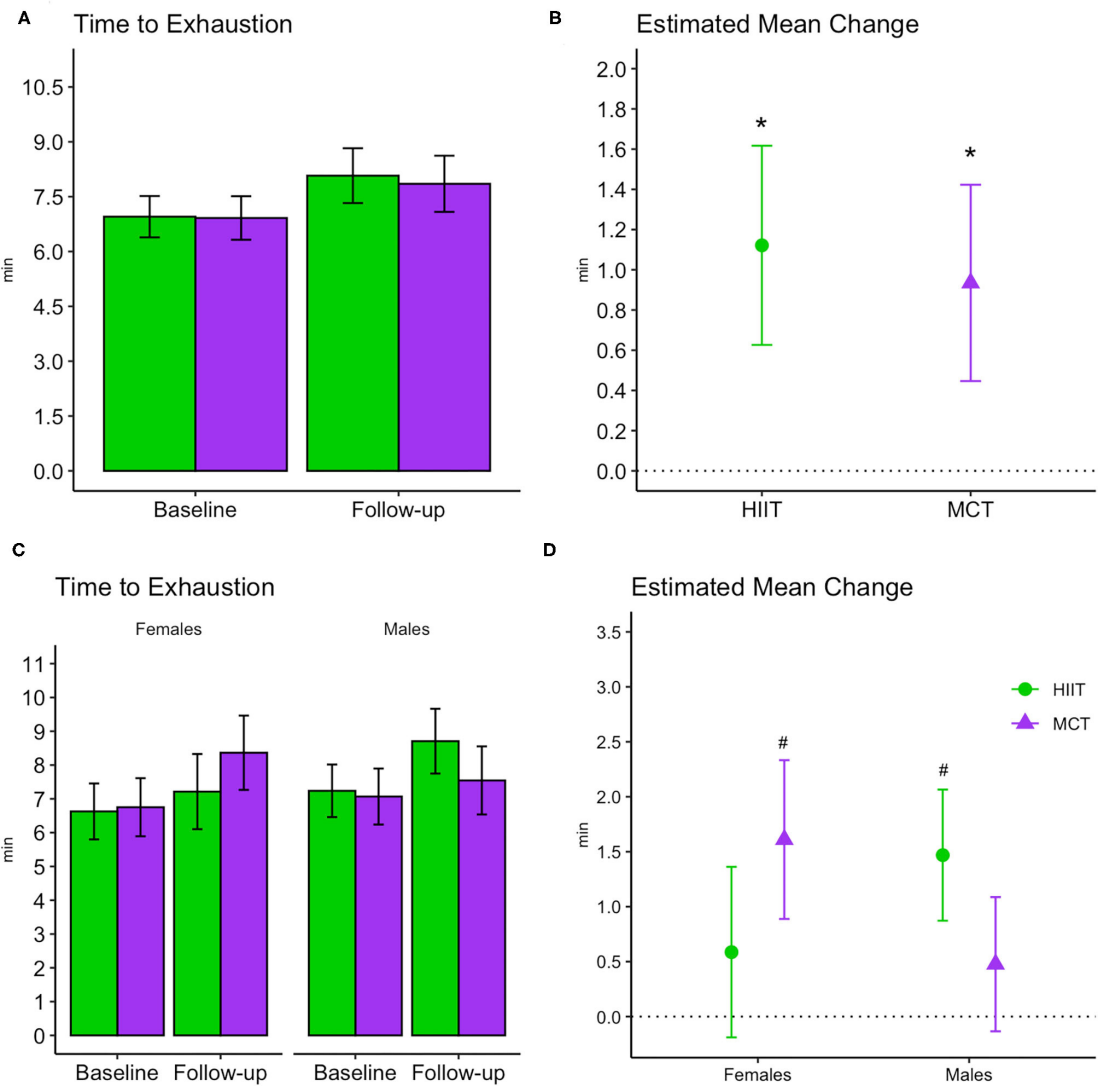
Baseline and follow-up means for time to exhaustion during exercise stress tests (bar plots, group mean and 95% CI) and estimated mean change from baseline to 6 months (scatterplots, estimated mean change and 95% CI). **(A,B)** Display changes for the entire sample, while **(C,D)** display changes stratified by males and females. *, significant changes over time; #, significant subgroup changes over time at adjusted significance threshold (*p* ≤ 0.005), with improvements in females within the MCT group [estimated mean change (95% CI): 1.61 min (0.9 to 2.33), *p* = 0.00003] and males within the HIIT group [1.47 min, (0.87 to 2.07), *p* = 0.000006]. See Supplementary Table 3 for further details. HIIT, high-intensity interval training; MCT, moderate-intensity continuous training.

Cardiorespiratory fitness improved in both groups at follow-up as demonstrated by a greater time to exhaustion [*F*_(1, 69)_ = 34.795, *p* < 0.001, Figure 7] and METs achieved [*F*_(1, 69.84)_ = 22.303, *p* < 0.001]; however, no between-group differences were noted. Results remained significant in fully adjusted models for time to exhaustion [*F*_(1, 69.13)_ = 35.985, *p* < 0.001] and METs achieved [*F*_(1, 72.87)_ = 21.841, *p* < 0.001]. Subgroup analyses revealed that significant changes in time to exhaustion were driven by greater improvements among females within the MCT group and males within the HIIT group at adjusted significance threshold (*p* ≤ 0.005, see Figure 7). Also, within group improvements at 6 months were driven mainly by enhanced performance on participant with greater fitness at baseline, see Supplementary Table 4).

### Adverse Events

We documented 12 study-related adverse events, 6 in the HIIT group and 6 in the MCT group. These adverse events were low-back pain (5), hip soreness (2), hypertensive crisis (1), knee soreness (2), and muscle soreness (2), and were resolved within the duration of the study.

## Discussion

The goal of our study was to investigate the impact of a 6-month HIIT with mind-motor training intervention on vascular and cognitive outcomes, compared to an active control group, in older adults with history of hypertension and SCD.

The effectiveness of exercise to improve cognition in those with hypertension remains to be determined, as there is dearth of RCTs on aerobic exercise to improve cognition in older adults with hypertension. Pierce and colleagues conducted a 4-month RCT comparing the effects of aerobic exercise on measures of executive function, memory, and processing speed in hypertensive young and older adults (29–59 years of age) compared to strength training, or a wait-list control group (Pierce et al., 1993). No differences between groups were observed after the intervention in any outcome. In an RCT administering a 4-month, multi-domain lifestyle intervention (i.e., diet, exercise and caloric restriction) to young older adults with hypertension, Smith and colleagues reported that those who engaged in an exercise program in addition to diet and caloric restriction improved executive function, memory and learning compared to a usual care control group, with positive effects also in VO_2_max and BP (Blumenthal et al., 2010; Smith et al., 2010). Taken together, these findings may suggest an additive benefit of aerobic exercise to cognition in adults with hypertension, however, due to fairly young samples involved in both RCTs and the uncertainty about the cognitive status of participants at baseline, their results may not be generalized to an older population of persons with hypertension and SCD. Also, these investigations only applied exercise protocols of MCT therefore, the effects of HIIT on cognition in this population has yet to be determined.

Our study is the first, to our knowledge, to investigate the impact of HIIT and mind-motor training on cognition and BP in older adults with a history of hypertension and SCD. Our assumptions were that by targeting BP control with HIIT and potentially amplifying cognitive improvements with supplementary mind-motor training, we would observe greater synergistic benefits to cognition. Strong evidence supports aerobic exercise as an effective therapy to lowering BP and managing hypertension (Pescatello et al., 2004). We hypothesized that these effects would, in turn, alleviate cerebrovascular burden and yield cognitive improvements and/or prevent decline (Baumgart et al., 2015). Despite lack of changes of GCF, our program did positively impact lower-level cognitive functioning measured via the TMT A and B, likely resulting from improved processing speed in both groups. Nonetheless, the more complex TMT set-shifting score (Part A-B) did not improve over time (Varjacic et al., 2018).

Our results indicated that the exercise program did not have the hypothesized effect on the study primary outcomes. At the end of the study, GCF remained unchanged despite improvements in cardiorespiratory fitness. Very few studies have conducted a similar investigation, with one trial reporting no changes in cognition in hypertensive middle-aged adults (Pierce et al., 1993). Therefore, it is plausible that our null findings on GCF signifies that hypertensive older adults with SCD may be less responsive to the benefits of HIIT and mind-motor training in higher-level cognitive functioning. This is possibly due to greater severity of hypertension burden on brain structure and function, which is prevalent in frontal-cortical and subcortical regions (Dichgans and Leys, 2017; Alber et al., 2019). This is reasonable considering that HIIT seems to impart benefits on memory in otherwise healthy older adults, as reported by Kovacevic et al. (2019) following a similar HIIT protocol. Another plausible explanation is that HIIT may not in fact be superior to MCT when assessing a range of different outcomes, including cognition, in older adults with a history of hypertension. Considering the recent findings of a systematic review and meta-analysis (Malmberg Gavelin et al., 2020), where small to medium effect sizes were observed for combined sequential exercise and cognitive training on cognition, it is also plausible that our study was underpowered to find significant effects.

We also reported no effects on SBP within or between groups. This finding was surprising and does not align with previous research on exercise and BP control in hypertensive patients (Molmen-Hansen et al., 2012; Pescatello et al., 2015). A plausible explanation for our BP results is that our sample included individuals with controlled and uncontrolled hypertension at study entry, which could have led to mixed response to our training protocol. Another investigation showed blunted SBP response in older adults with controlled hypertension following HIIT and MCT (Iellamo et al., 2014). Molmen-Hansen et al. (2012) reported significant SBP reduction in middle-aged adults following a similar HIIT protocol, and their sample only included individuals with uncontrolled BP at baseline. Our subgroup analysis offered confirmation to this hypothesis with HIIT having the greatest effect on participants with high BP at study entry compared to individuals with low BP across groups.

Despite lack of changes in SBP, we reported reduction in DBP following the program within the HIIT group. Noteworthy, 9 participants reported changes in their BP medication throughout the study. We repeated our analysis excluding these participants and DBP changes remained significant within the HIIT group, which may strengthen our results. These findings may hint at a specific positive impact of HIIT on DBP control in this population, similar to the results by Iellamo et al. (2014); however, this can be considered a small effect size and more conclusive evidence is warranted. As such, even though our findings and previous literature suggest HIIT could positively impact DBP to greater extent in hypertensive older adults, while also possibly improving SBP in those with uncontrolled BP (Molmen-Hansen et al., 2012; Iellamo et al., 2014), HIIT and MCT may in fact have similar effects in this population. Furthermore, the extent to which these potential improvements will reflect cognitive enhancements remains to be determined.

It is possible that the high attrition rate and low adherence to the exercise sessions may have hindered greater effects of our program. It is plausible that these shortcomings were a result of the demands of taking part in an exercise program centered at guaranteeing that participants complied with the exercise intensities prescribed. We used objective and subjective measures of exercise intensity, including immediate continuous feedback, to ensure compliance with the training protocols. These considerations, however, remain speculative and these shortcomings should be carefully considered in future exercise studies with this population.

## Limitations

We included individuals with controlled and uncontrolled hypertension, and did not account for the effects of diet, smoking, alcohol intake and physical activity levels (Gottesman et al., 2017). Adjusting for these factors could have impacted study results, especially past physical activity levels and sedentary time—considering that these are modifiable risk factors for cardiovascular disease and have been shown to impact cognition across the life span (Falck et al., 2017). The usage of BP medication that decreases HR in this population also poses a challenge for accurately prescribing exercise intensity based on %HRmax—hence, RPE was also used to mitigate this issue in the current study. Furthermore, approximately 84% of participants achieved 80% or more of their age-predicted HRmax (208 – 0.7 × age) (Tanaka et al., 2001) at baseline, suggesting that any HR-lowering effect of BP medication did not have a substantial impact on our sample. We also noted a trend for differences between groups in history of depression (Table 1), with the MCT group including more patients with this condition. Adjusting for history of depression in the models showed that it had a significant contribution to models of cardiorespiratory fitness (all *p* < 0.017), however it did not change overall study results. Future studies should consider exploring this relationship of cardiorespiratory fitness improvements and depression in older adults with hypertension.

Also, although the CBS cognitive battery is grounded in well-validated neuropsychological tests (Hampshire et al., 2012), it has not been widely used in exercise studies and it may lack sensitivity in our clinical sample. That said, this seems unlikely, because the tasks have previously been shown to be highly sensitive to subtle cognitive differences related to disease or pharmacological intervention. For example, the test of planning (the Hampshire Tree Task) is sensitive to performance differences between specific genotypes in early Parkinson's disease (Williams-Gray et al., 2007); tests of paired-associates learning, such as the one employed in this study, are able to distinguish between first-episode schizophreniform psychosis and established schizophrenia (Wood et al., 2002) and the Token Search task used here has been used to detect increases in spatial working memory in children with attention deficit/hyperactivity disorder following a low dose of methylphenidate (Mehta et al., 2000).

We were unable to collect neuroimaging or biomarker data in this trial, and accordingly, this limits our ability to fully assess the impact of HIIT compared to MCT on underlying mechanisms for cognitive improvement. As well, another potential limitation is that we did not include a non-exercising control group in the current study. The lack of such control group poses a challenge to determine whether our results reflect the effects of extrinsic mechanisms, especially for cognition. Aerobic-based exercise does appear to have a positive effect on cognition compared to controls, and is already recommended as a non-pharmacological approach to mitigate dementia risk (Livingston et al., 2020; Yu et al., 2020). Meanwhile, our overarching goal was to determine whether exercising at higher intensity would be superior to a moderate intensity intervention. That is, modulating key elements of an intervention to further refine exercise prescription in clinical populations at risk of dementia, as we have done in the past (Gill et al., 2016; Gregory et al., 2017; Heath et al., 2017; Shellington et al., 2017; Boa Sorte Silva et al., 2018, 2020b). As such, the inclusion of non-exercising control group was not within the scope of the current study.

Lastly, participants were predominantly Caucasian, highly educated and functionally independent, limiting generalizability of our findings. Exercise sessions for the final wave of participants ended 3 weeks earlier due to closures caused by the COVID-19 pandemic, which also prevented 13 participants from attending their final assessment (impacting our attrition rate).

## Future Directions

Future studies should emphasize comprehensive multidomain interventions for individuals with hypertension and SCD. This is relevant since pharmacological therapies to treat hypertension do not seem to reduce dementia risk (Williamson et al., 2019). Emerging evidence suggests synergistic effects on cognition and SBP as a result of exercise, a healthy diet and weight management in hypertensive middle-aged adults (Smith et al., 2010). Replicating these findings in older adults with SCD and hypertension could allow refinement of lifestyle interventions to reduce dementia risk.

## Conclusions

In this trial involving community-dwelling older adults with history of hypertension and SCD, aerobic exercise of either high or moderate-intensity, combined with mind-motor training, did not improve cognition or SBP, despite improvements in cardiorespiratory fitness and lower-level cognitive functioning.

## Data Availability Statement

Data will be made available upon reasonable request. Requests to access the datasets should be directed to robert.petrella@ubc.ca.

## Ethics Statement

The studies involving human participants were reviewed and approved by Western University Health Sciences Research Ethics Board. The patients/participants provided their written informed consent to participate in this study.

## Author Contributions

NB contributed to study concept and design, recruitment, implementation, data management, data analysis, interpretation of results, and drafted the manuscript. AP, CM, and NC contributed to recruitment, implementation, data management, interpretation of results, and critical review of the manuscript. DG contributed to study concept and design, data analysis, interpretation of results, and critical review of the manuscript. AO contributed to study design, interpretation of results, and critical review of the manuscript. RP contributed to study concept and design, recruitment, implementation, data management, data analysis, interpretation of results, critical review of the manuscript, and secured funding for the study. All authors contributed to the article and approved the submitted version.

## Conflict of Interest

The cognitive tests used in this study are marketed by Cambridge Brain Sciences, of which AO is the Chief Scientific Officer. Under the terms of the existing licensing agreement, AO and his collaborators are free to use the platform at no cost for their scientific studies, and such research projects neither contribute to, nor are influenced by, the activities of the company. Consequently, there is no overlap between the current study and the activities of Cambridge Brain Sciences, nor was there any cost to the authors, funding bodies, or participants who were involved in the study. The authors declare that the research was conducted in the absence of any commercial or financial relationships that could be construed as a potential conflict of interest.
