# Supplementary material for: The Benefits of High-Intensity Interval Training on Cognition and Blood Pressure in Older Adults With Hypertension and Subjective Cognitive Decline: Results From the Heart & Mind Study
Source: Front Aging Neurosci. 2021 Apr 15;13:643809. doi: 10.3389/fnagi.2021.643809 (PMC8082143; doi:10.3389/fnagi.2021.643809)
Supplement: Supplementary file 1 [file Table_1.DOCX]

**SUPPLEMENTAY MATERIAL**

**Supplementary Methods**

Description of the domain-specific composite scores derived from the Cambridge Brain Sciences cognitive battery tasks.

*Memory*

Composite measure derived from the Cambridge Brain Sciences cognitive battery (Hampshire et al., 2012). The composite score is derived from 4 cognitive tasks: Monkey Ladder, Spatial Span, Digit Span, and Paired Associates. For each of the cognitive tasks, higher scores indicate better outcomes. The scores from each task are standardized and averaged to generate a standardized composite measure, i.e., z scores with a mean of 0 and a standard deviation of 1.

Below is the description of each individual task.

1. **Monkey Ladder** is a based on a task from the animal literature (non-human primate) and assesses working memory ability (Inoue and Matsuzawa, 2007). In this task, sets of numbered boxes are displayed all at the same time at random locations within a grid. After a variable interval (number of boxes multiplied by 900 ms), the numbers are removed leaving just the blank boxes visible. Participants are requested to respond by clicking on the boxes in ascending numerical sequence. The difficulty of the task is modulated as follows: the number of boxes presented increases by one if participant answers correctly and decreases by one if participant makes a mistake. After three mistakes, the test ends. The outcome measure is the length of the longest sequence successfully remembered.
2. **Spatial Span** is a task to measure spatial short-term memory capacity in humans (Kessels et al., 2000). In this task, 16 boxes are displayed in a grid. A sequence of randomly selected boxes flashes one at a time at a rate of 900 ms per box. Subsequently, a tone cues the participant to repeat the sequence by clicking on the boxes in the same order in which they flashed. The difficulty of the task is modulated as follows: the number of boxes that flash increases by one if participant answers correctly and decreases by one if participant makes a mistake. After three mistakes, the test ends. The outcome measure is the length of the longest sequence successfully remembered.
3. **Digit Span** is based on the verbal working memory component of the WAIS-R intelligence test (Wechsler, 1981). In this task, participants view a sequence of digits that appear on the screen one at a time. Subsequently, participants are required to repeat the sequence of numbers by using the mouse cursor to click a series of numbered buttons that appear along the bottom of the screen. The difficulty of the task is modulated as follows: the sequence of numbers on the screen increases by one if participant answers correctly and decreases by one if participant makes a mistake. After three mistakes, the test ends. The outcome measure is the length of the longest digit sequence successfully remembered.
4. **Paired Associates** is a visuospatial paired associate learning task (Gould et al., 2005). In this task, boxes are displayed at random locations on a grid. The boxes open one after another to reveal an enclosed icon, after which they close. Subsequently, the icons are displayed in random order in the centre of the grid and the participant must click on the boxes that contained them. The difficulty of the task is modulated as follows: if the participant remembers all the icon-location pairs correctly, then the next trial will have one more box. If a mistake is made, the next trial has one less box. After three mistakes, the test ends.

*Reasoning*

Composite measure from the Cambridge Brain Sciences cognitive battery. The composite score is derived from 3 cognitive tasks: Grammatical Reasoning, Double Trouble, and Odd One Out. For each of the cognitive tasks, higher scores indicate better outcomes. The scores from each task are standardized and averaged to generate a standardized composite measure, i.e., z scores with a mean of 0 and a standard deviation of 1.

Below is the description of each individual task:

1. **Grammatical Reasoning** is a test based on Baddeley’s 3-minute grammatical reasoning test (Baddeley, 1968). In this task, a written statement appears on the screen and the participant must indicate whether the statement correctly describes the relationship between two shapes displayed below, by indicating “True” or “False”. The participant has 90 seconds to complete as many trials as possible. A correct answer increases the total score by one point, while a wrong answer decreases the score by one point. The outcome measure is the total score at the end of the test.
2. **Double Trouble** is a new version of the Stroop test (Stroop, 1935). In this task, a coloured word is displayed at the top of the screen (e.g., the word RED drawn in blue ink). Participants must indicate which of two-coloured words at the bottom of the screen describes the colour that the word at the top of the screen is drawn in. The colour word mappings may be congruent, incongruent, or doubly incongruent, depending on whether or not the colours that a given word describes matches the colour that it is drawn in. The participant has 90 seconds to complete as many trials as possible. A correct answer increases the total score by one point, while a wrong answer decreases the score by one point. The outcome measure is the total score at the end of the test.
3. **Odd One Out** is based on a subgroup of problems from the Cattell Culture Fair Intelligence Test (Cattell, 1943) designed to measure deductive reasoning. In this task, nine groups of coloured shapes appear in a grid. The features that define the shapes (colour, shape, number of copies) are related to each other according to a set of rules. The participant must deduce the rules that relate the object features and select the one option whose contents do not correspond to those rules. The score is the number of total correct answers. The participant has 90 seconds to complete as many trials as possible and the puzzles progressively increase in difficulty. A correct answer increases the total score by one point, while a wrong answer decreases the score by one point. The outcome measure is the total score at the end of the test.

*Planning*

Composite measure from the Cambridge Brain Sciences cognitive battery. The composite score is derived from 2 cognitive tasks: Spatial Planning and Token Search. For each of the cognitive tasks, higher scores indicate better outcomes. The scores from each task are standardized and averaged to generate a standardized composite measure, i.e., z scores with a mean of 0 and a standard deviation of 1.

Below is the description of each individual task.

1. **Spatial Planning** is based on the Tower of London Task and was designed to assess planning and executive function (Shallice, 1982). In this task, nine numbered beads are positioned on a tree shaped frame. The participant repositions the beads one-by-one so that they are configured in ascending numerical order running from left to right and top to bottom of the tree. The participant has 3 minutes to complete as many trials as possible and the puzzles progressively increase in difficulty—requiring more moves and more complex planning. The trial ends if the participant takes more than the double of the number of moves necessary to solve the problem. A successfully solved puzzle increases the final score according to this formula: (2 x minimum number of moves required) – moves made. The outcome measure is the total score after 3 minutes.
2. **Token Search** is a task commonly used to measure strategy during search behaviour (Collins et al., 1998) and is believed to assess working memory and strategy. In this task, sets of boxes are displayed on the screen in random locations in a grid, a token is hidden in one of the boxes. The participant must find the hidden token by clicking on the boxes one at a time. When the token is found, it is hidden within another box. The token will not appear within the same box twice, therefore, the participant must search the boxes until the token has been found once within each box. A mistake is made if the participant clicks on a box that has either already been clicked while trying to find the token, or previously contained the token. The difficulty of the task is modulated as follows: if a mistake is made, a new trial will begin with one less box, while if the token is found once in each box without any errors, a new trial will begin with one more box. After three mistakes, the test ends. The outcome measure is the maximum level completed.

*Concentration*

Composite measure from the Cambridge Brain Sciences cognitive battery. The composite score is derived from 3 cognitive tasks: Rotations, Feature Match Task, and Polygons. For each of the cognitive tasks, higher scores indicate better outcomes. The scores from each task are standardized and averaged to generate a standardized composite measure, i.e., z scores with a mean of 0 and a standard deviation of 1.

Below is the description of each individual task.

1. **Rotations** is a task that measures the ability to spatially manipulate objects in mind (Silverman et al., 2000). In this variant, two groups of coloured squares are displayed to either side of the screen with one of the groups rotated by a multiple of 90 degrees. Each group is a number of N squares. The groups are either identical (when unrotated) or differ by the position of at least one of them. The participant must indicate whether the groups are identical or different, solving as many problems as possible in 90 seconds. A correct response increases the final score by N, and the subsequent trial has groups of N + 1 squares. If a mistake is made, the total score decreased by N, and the next trial has groups of N – 1 squares. The outcome measure is the total score.
2. **Feature Match** is a task based on feature search tasks applied to assess attentional processing (Treisman and Gelade, 1980). In this task, two groups of items are displayed on the screen, each containing a number N of abstract shapes. The groups are either identical or different in the item features (and item position) and participant must indicate whether both groups match (if identical) or mismatch (if different). A correct response increases the final score by N, and the subsequent trial has groups of N + 1 items. If a mistake is made, the total score decreased by N, and the next trial has groups of N – 1 items. The outcome measure is the total score.
3. **Polygons** is based on the Interlocking Pentagons task, part of the Mini-Mental Status Examination (Folstein et al., 1975). In this task, a pair of wire-framed overlapping polygons is displayed on one side of the screen. The participant must indicate whether a polygon displayed on the other side of the screen is identical to one of the interlocking polygons, solving as many problems as possible in 90 seconds. If responses are correct, the total score increases by the difficulty level and the differences between the polygons becomes increasingly subtle. If the responses are incorrect, the total score decreases by the difficulty level and the difference between the polygons become more pronounced. The outcome measure is the total score. Supplementary references

**References**

Baddeley, A. D. (1968). A 3 min reasoning test based on grammatical transformation. *Psychon. Sci.* 10, 341–342. doi:10.3758/BF03331551.

Cattell, R. B. (1943). The measurement of adult intelligence. *Psychol. Bull.* 40, 153–193. doi:10.1037/h0059973.

Collins, P., Roberts, A. C., Dias, R., Everitt, B. J., and Robbins, T. W. (1998). Perseveration and strategy in a novel spatial self-ordered sequencing task for nonhuman primates: Effects of excitotoxic lesions and dopamine depletions of the prefrontal cortex. *J. Cogn. Neurosci.* 10, 332–354. doi:10.1162/089892998562771.

Folstein, M. F., Folstein, S. E., and McHugh, P. R. (1975). “Mini-mental state”. A practical method for grading the cognitive state of patients for the clinician. *J. Psychiatr. Res.* 12, 189–198. doi:10.1016/0022-3956(75)90026-6.

Gould, R. L., Brown, R. G., Owen, A. M., Bullmore, E. T., Williams, S. C. R., and Howard, R. J. (2005). Functional neuroanatomy of successful paired associate learning in Alzheimer’s disease. *Am. J. Psychiatry* 162, 2049–2060. doi:10.1176/appi.ajp.162.11.2049.

Hampshire, A., Highfield, R. R., Parkin, B. L., and Owen, A. M. (2012). Fractionating Human Intelligence. *Neuron* 76, 1225–1237. doi:10.1016/j.neuron.2012.06.022.

Inoue, S., and Matsuzawa, T. (2007). Working memory of numerals in chimpanzees. *Curr. Biol.* 17, R1004–R1005. doi:10.1016/j.cub.2007.10.027.

Kessels, R. P. C., Van Zandvoort, M. J. E., Postma, A., Kappelle, L. J., and De Haan, E. H. F. (2000). The Corsi Block-Tapping Task: Standardization and normative data. *Appl. Neuropsychol.* 7, 252–258. doi:10.1207/S15324826AN0704_8.

Shallice, T. (1982). Specific impairments of planning. *Philos. Trans. R. Soc. Lond. B. Biol. Sci.* doi:10.1098/rstb.1982.0082.

Silverman, I., Choi, J., Mackewn, A., Fisher, M., Moro, J., and Olshansky, E. (2000). Evolved mechanisms underlying wayfinding: further studies on the hunter-gatherer theory of spatial sex differences. *Evol. Hum. Behav.* 21, 201–213. doi:10.1016/S1090-5138(00)00036-2.

Stroop, J. R. (1935). Studies of interference in serial verbal reactions. *J. Exp. Psychol.* 18, 643–662. doi:10.1037/h0054651.

Treisman, A. M., and Gelade, G. (1980). A feature-integration theory of attention. *Cogn. Psychol.* 12, 97–136. doi:10.1016/0010-0285(80)90005-5.

Wechsler, D. (1981). The psychometric tradition: Developing the wechsler adult intelligence scale. *Contemp. Educ. Psychol.* 6, 82–85. doi:10.1016/0361-476X(81)90035-7.

**Supplementary Table 1.** Complete-case and multiple-imputation data analysis of study primary outcomes.

|  | Within-group estimated mean change (95% CI) | | | | Between-group differences (95% CI) | |
| --- | --- | --- | --- | --- | --- | --- |
| Outcomes ^a^ | HIIT | *p* Value | MCT | *p* Value | 6 months | *p* Value |
| Systolic blood pressure |  |  |  |  |  |  |
| Complete-case ^b^ | -1.11 (-5.35 to 3.13) | 0.60 | 0.62 (-3.45 to 4.69) | 0.76 | -1.73 (-7.6 to 4.15) | 0.56 |
| Multiple-imputation ^c^ | -0.54 (-5.02 to 3.94) | 0.81 | 1.18 (-2.83 to 5.19) | 0.56 | -1.72 (-7.23 to 3.78) | 0.54 |
| Global cognitive functioning |  |  |  |  |  |  |
| Complete-case ^b^ | -0.03 (0.13 to 0.08) | 0.61 | 0.05 (-0.05 to 0.15) | 0.31 | -0.08 (-0.22 to 0.06) | 0.28 |
| Multiple-imputation ^c^ | -0.01 (-0.11 to 0.08) | 0.77 | 0.05 (-0.04 to 0.15) | 0.27 | -0.07 (-0.19 to 0.06) | 0.28 |

^a^ Calculated from linear mixed effects regression models that included group (HIIT or MCT), time (baseline and 6 months), and group × time interaction terms.

^b^ Data available from 75 participants who completed both baseline and follow-up assessment (HIIT = 36, MCT = 39).

^c^ Pooled statistics including 40 multiple-imputation datasets (HIIT = 65, MCT = 63).

Notes: CI = confidence interval; HIIT = high-intensity interval training; MCT = moderate intensity.

**Supplementary Table 2**. Results for baseline resting blood pressure subgroups.

| Outcomes ^a^ | HIIT (n = 65) | | | | MCT (n = 63) | | | | Subgroup × time interaction |
| --- | --- | --- | --- | --- | --- | --- | --- | --- | --- |
|  | High BP (n = 32) | *p* Value | Low BP (n = 33) | *p* Value | High BP (n = 32) | *p* Value | Low BP (n = 31) | *p* Value |  |
| Systolic blood pressure | **-8.96 (-14.78 to -3.14)*** | **0.003** | 3.18 (-2.08 to 8.43) | 0.23 | -2.79 (-8.04 to 2.47) | 0.29 | 5.32 (-0.07 to 10.7) | 0.053 | **F_(3,74.57)_ = 5.24,**  **p = 0.002** |
| Global cognitive functioning | -0.02 (-0.16 to 0.13) | 0.83 | -0.03 (-0.16 to 0.1) | 0.68 | 0.19 (0.06 to 0.32) | 0.006 | -0.07 (-0.21 to 0.06) | 0.27 | F_(3, 72.96)_ = 3.05,  p = 0.034 |
| Time to exhaustion | **1.41 (0.66 to 2.16)** | **0.0004** | 0.91 (0.26 to 1.56) | 0.007 | 0.57 (-0.13 to 1.28) | 0.11 | **1.26 (0.59 to 1.93)** | **0.0004** | F_(3,67)_ = 1.09,  p = 0.36 |

^a^ Data presented as estimated mean change (95% confidence interval).

Notes: Bold numbers indicate significant results at adjusted significance threshold (p ≤ 0.005).

* = greater changes compared to HIIT Low BP (difference between groups [CI]: -12.14 mmHg [-19.98 to -4.30], p = 0.003) and MCT Low BP (-14.28 mmHg [-22.22 to -6.35], p = 0.001) adjusted significance threshold (p ≤ 0.005).

HIIT = high-intensity; MCT = moderate-intensity continuous training.

**Supplementary Table 3.** Results for biological sex subgroups.

| Outcomes ^a^ | HIIT (n = 65) | | | | MCT (n = 63) | | | | Subgroup × time interaction |
| --- | --- | --- | --- | --- | --- | --- | --- | --- | --- |
|  | Males (n = 33) | *p* Value | Females (n = 32) | *p* Value | Males (n = 34) | *p* Value | Females (n =29) | *p* Value |  |
| Systolic blood pressure | 0.01 (-5.27 to 5.30) | 1.00 | -5.71 (-12.12 to 0.69) | 0.08 | 2.19 (-3.18 to 7.56) | 0.42 | -0.06 (-5.86 to 5.74) | 0.98 | F_(3, 79.77)_ = 1.23  p = 0.31 |
| Global cognitive functioning | -0.01 (-0.14 to 0.12) | 0.84 | -0.03 (-0.19 to 0.13) | 0.70 | 0.004 (-0.13 to 0.14) | 0.96 | 0.12 (-0.02 to 0.27) | 0.90 | F_(3, 72.81)_ = 0.93,  p = 0.43 |
| Time to exhaustion | **1.47 (0.87 to 2.07)** | **0.000006** | 0.59 (-0.19 to 1.36) | 0.14 | 0.48 (-0.13 to 1.09) | 0.12 | **1.61 (0.89 to 2.33)** | **0.00003** | F_(3, 67.07)_ = 3.1,  p = 0.033 |

^a^ Data presented as estimated mean change (95% confidence interval).

Notes: Bold numbers indicate significant changes over time at adjusted significance threshold (p ≤ 0.005).

HIIT = high-intensity interval training; MCT = moderate-intensity continuous training.

**Supplementary Table 4.** Results for baseline cardiorespiratory fitness subgroups.

| Outcomes ^a,b^ | HIIT (n = 62) | | | | MCT (n = 56) | | | | Subgroup × time interaction |
| --- | --- | --- | --- | --- | --- | --- | --- | --- | --- |
|  | High FIT (n = 30) | *p* Value | Low FIT (n = 32) | *p* Value | High FIT (n = 29) | *p* Value | Low FIT (n =27) | *p* Value |  |
| Systolic blood pressure | -0.87 (-6.36 to 4.61) | 0.75 | -3.89 (-10.15 to 2.37) | 0.22 | 0.05 (-5.35 to 5.45) | 0.99 | 2.23 (-3.96 to 8.4) | 0.48 | F_(3, 77.54)_ = 0.66,  p = 0.58 |
| Global cognitive functioning | 0.02 (-0.11 to 0.15) | 0.76 | -0.09 (-0.24 to 0.07) | 0.27 | 0.05 (-0.08 to 0.18) | 0.46 | 0.06 (-0.09 to 0.22) | 0.41 | F_(3, 71.73)_ = 0.79,  P = 0.51 |
| Time to exhaustion | **1.40 (0.76 to 2.04)** | **0.00004** | 0.79 (0.01 to 1.58) | 0.047 | **0.93 (0.30 to 1.55)** | **0.004** | 1.00 (0.21 to 1.78) | 0.013 | F(3, 67.43) = 0.60, p = 62. |

^a^ Data presented as estimated mean change (95% confidence interval).

^b^ Data available from 118 (HIIT = 62, MCT = 56).

Notes: Bold numbers indicate significant changes over time at adjusted significance threshold (p ≤ 0.005).

HIIT = high-intensity interval training; MCT = moderate-intensity continuous training; High FIT = high fitness at baseline; Low FIT = low fitness at baseline.
